# Robotic-assisted gastrointestinal stromal tumor (GIST) resection with endoscopic transoral specimen retrieval (Gastrointestinal Cancer-NOSES Type IX): a case report and literature review

**DOI:** 10.3389/fonc.2025.1580558

**Published:** 2025-04-29

**Authors:** Lang Wang, Jing Zhang, Dehai Wang, Yifeng Zang, Xianglin Zhu, Shijun Zhao, Cheng Zhao, Hao Liang, Jie Zhang, Yinlu Ding

**Affiliations:** Department of General Surgery, The Second Hospital of Shandong University, Cheeloo College of Medicine, Shandong University, Jinan, China

**Keywords:** robotic-assisted surgery, gastrointestinal stromal tumor (GIST), transoral specimen retrieval, natural orifice specimen extraction surgery (NOSES), GC-NOSES Type IX

## Abstract

**Objective:**

To investigate the methodology and outcomes of Da Vinci robotic-assisted resection of gastrointestinal stromal tumors (GISTs) combined with endoscopic transoral specimen retrieval (GC-NOSES type IX), establishing a benchmark for minimally invasive treatment of GISTs.

**Methods:**

This manuscript details a case involving a GIST situated on the posterior wall of the distal gastric body, adjacent to the lesser curvature, with a size of approximately 2.7 cm and exhibiting an intraluminal growth pattern. The tumor was effectively excised through robot-assisted GIST resection, complemented by endoscopic transoral specimen extraction (GC-NOSES type IX). The case is analyzed alongside pertinent literature and surgical perspectives.

**Results:**

The patient was admitted with “persistent abdominal discomfort persisting for over two months.” Preoperative enhanced abdominal CT reveals a gastric body lesion measuring approximately 2.7 centimeters, suggestive of a GIST. The patient underwent a successful robot-assisted resection of the GIST, with endoscopic transoral specimen extraction (GC-NOSES type IX). Postoperative histopathological analysis confirmed a GIST measuring 4.0 cm × 3.0 cm × 3.0 cm, classified as low-risk, with clear resection margins. Immunohistochemical profiling showed CD117 (+), CD34 (+), Desmin (-), DOG-1 (+), Ki67 (approximately 5% positive tumor cells), S-100 (-), SDHB (+), SMA (a few cells +), and SOX-10 (-).

**Conclusion:**

GISTs are the most common mesenchymal tumors found in the gastrointestinal tract, with a predominant occurrence in the stomach. The primary treatment approach is R0 resection. There is a clear trend towards minimally invasive techniques. Robotic-assisted gastric GIST resection with endoscopic transoral specimen extraction (GC-NOSES type IX) has shown significant advantages in minimally invasive surgery. However, the esophagus’s unique anatomical structure necessitates careful selection of surgical indications, mastery of operative techniques, and excellent team coordination. Ensuring surgical safety is crucial to fully harness the minimally invasive benefits of this technique, thereby optimizing patient outcomes.

## 1 Introduction

Gastrointestinal stromal tumors (GISTs), originating from interstitial cells of Cajal, represent the predominant form of mesenchymal neoplasms, with approximately 70% of occurrences localized in the stomach (1, 2). With the progression of research into the biological properties of GIST, total surgical excision continues to be the foremost and most efficacious therapeutic approach, with routine lymphadenectomy being excluded (3, 4). Recent investigations into GIST surgery have primarily concentrated on the advancement of minimally invasive surgical methodologies and the implementation of enhanced recovery after surgery protocols (5). In comparison to traditional open surgery and laparoscopic techniques, the Da Vinci robotic system offers potential benefits by markedly improving surgical precision. Additionally, for patients with gastric GISTs situated in difficult-to-access locations, robot-assisted surgery may broaden the criteria for minimally invasive treatment of GISTs (6–8). During the surgical procedure, it is frequently required to enlarge the abdominal wall incision to aid in specimen extraction post-surgery, potentially increasing the risk of complications like surgical site infection and incisional hernia (9). Natural orifice specimen extraction surgery (NOSES) entails the retrieval of postoperative specimens through natural orifices, including the vagina, oral cavity, or rectum. This technique obviates the necessity for supplementary abdominal incisions, presenting an innovative strategy for minimally invasive interventions. Specifically, when addressing gastric tumors, the procedure utilizing oral extraction is classified as GC-NOSES IX, contingent upon the surgical methodology and extraction route (10). Currently, there is limited literature on the endoscopic biopsy of GISTs, and there are no documented reports regarding the use of the Da Vinci robotic system in conjunction with endoscopic techniques for the resection of gastric GISTs. Our department has executed over 500 robot-assisted gastrointestinal surgeries, bolstered by a team of endoscopists with substantial expertise. Furthermore, we have undertaken a series of robot-assisted NOSES procedures for gastrointestinal tumors, accruing considerable proficiency in robotic NOSES and dual-scope surgeries. Recently, our center successfully performed a transoral specimen retrieval and endoscopic resection of a GIST located in the posterior wall of the lower gastric body using the da Vinci robotic system without an abdominal auxiliary incision (GC-NOSES IX technique). This approach effectively integrates the advantages of the da Vinci surgical operating system with endoscopic techniques, significantly enhancing the overall postoperative quality of life for the patient. We present this case report.

## 2 Case report

### 2.1 Patient-derived data

**Figure 1:**
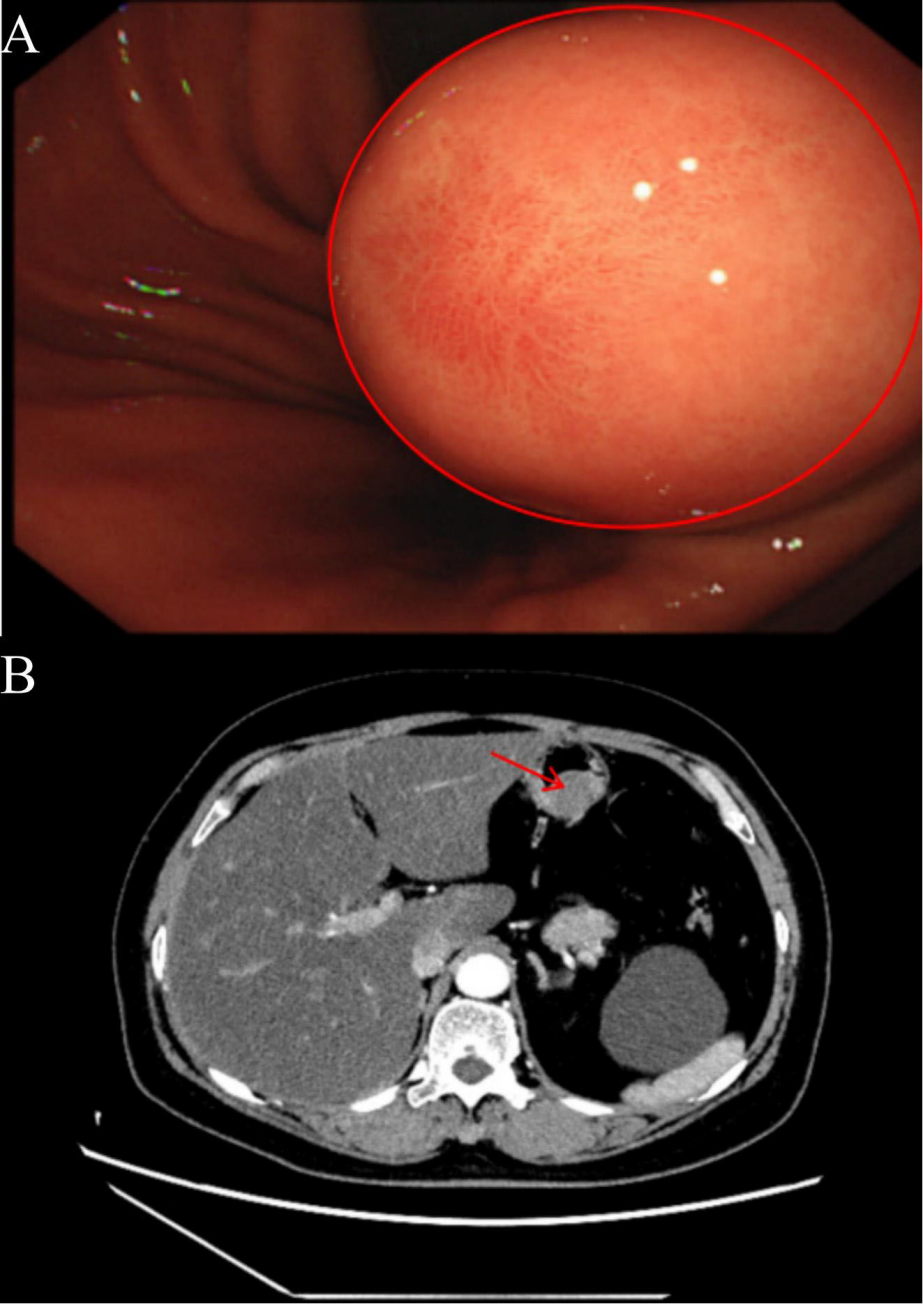
**(A)** The red circle indicates the gastroscopic image of the patient, revealing a significant submucosal mass in the posterior wall of the lower gastric body, characterized by limited mobility and a firm texture. **(B)** The red arrow indicates the tumor’s location in the upper abdomen as demonstrated by the enhanced CT scan, highlighting the arterial phase enhancement, with the tumor protruding into the gastric lumen.

The patient, a 61-year-old female with a body mass index of 32.9 kg/m², was admitted for persistent abdominal discomfort lasting over two months. Her medical history includes a surgical intervention for ectopic pregnancy over three decades ago and a four-year history of hypertension, effectively managed with amlodipine. Physical examination revealed significant tenderness in the upper abdomen, though no palpable masses were detected. Gastroscopy identified a substantial submucosal protrusion on the posterior wall of the lower gastric body, characterized by limited mobility and firm consistency (**Figure 1A**). An enhanced abdominal CT scan revealed a gastric body mass measuring approximately 2.7 cm in diameter, raising suspicion of a GIST (**Figure 1B**). Cardiac ultrasound, lower extremity deep vein ultrasound, and tumor markers revealed no significant abnormalities. Preoperative diagnosis includes: 1. gastric mass lesion 2. chronic non-atrophic gastritis 3. chronic inflammation of the esophageal squamous epithelium 4. fatty liver 5. hypertension 6. type 2 diabetes mellitus 7. simple left renal cyst 8. right pulmonary bulla 9. tracheal diverticulum 10. atherosclerosis of the aorta and coronary arteries 11. post-extraterrestrial pregnancy. Preoperative assessments indicate elevated fasting blood glucose; I recommend a consultation with the endocrinology and metabolism department to assist in stabilizing blood glucose levels. Additionally, a comprehensive multidisciplinary consultation should be conducted to rule out any surgical contraindications before considering the feasibility of the procedure.

### 2.2 Surgical Technique and Critical Procedures

#### 2.2.1 Positioning and Trocar Site Selection

**Figure 2:**
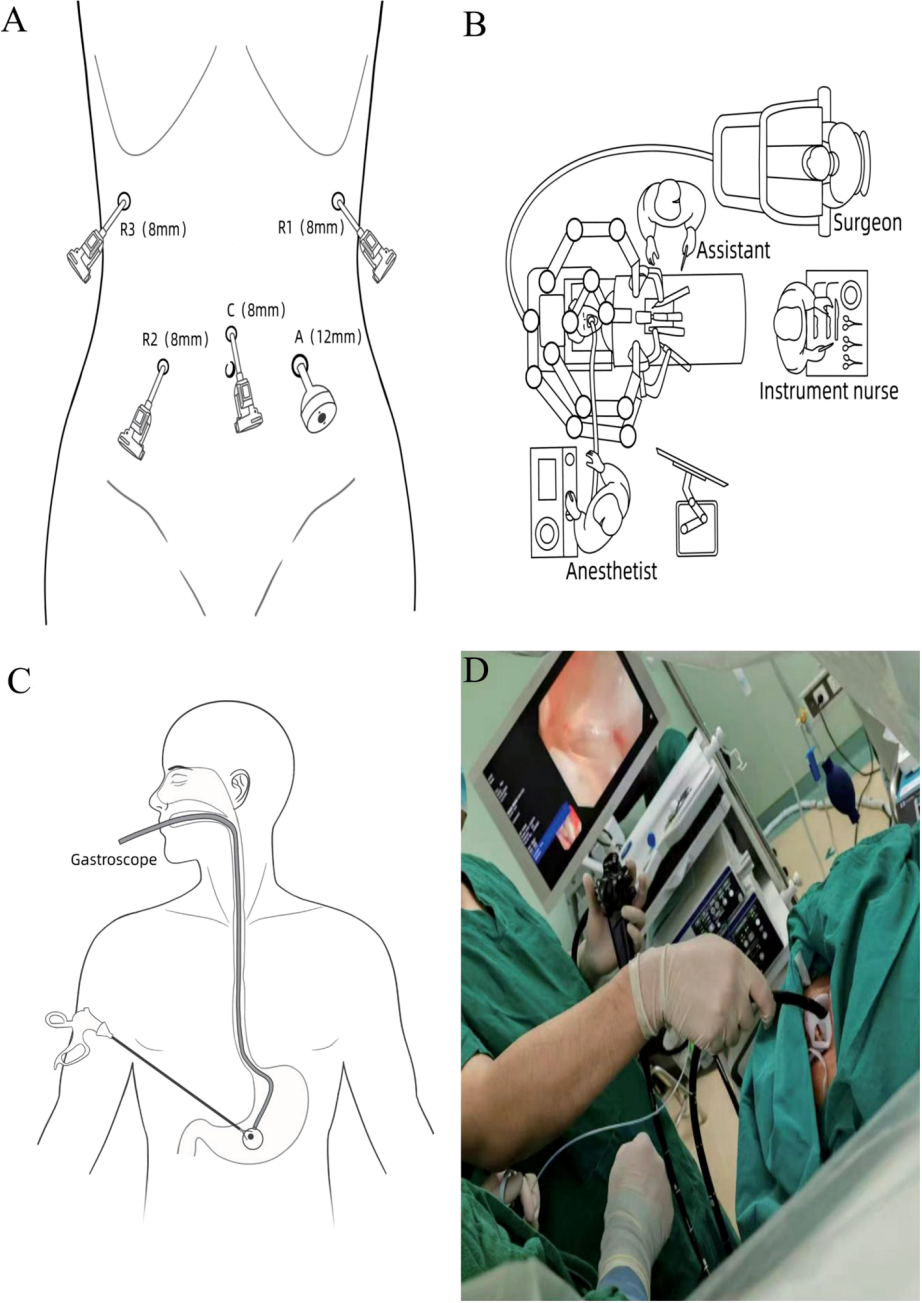
**(A)** Surgical port configuration. **(B)** Schematic representation of operating room positioning. **(C, D)** Illustration of specimen retrieval under gastroscopy.

Following successful endotracheal intubation and general anesthesia, the patient is placed in a supine position with legs spread apart. A urinary catheter is inserted, and deep venous access is established. After routine disinfection and draping, considering the patient’s history of ectopic pregnancy and the presence of a midline incision in the lower abdomen, a small incision is made 1 cm above the umbilicus. A Veress needle is utilized to establish pneumoperitoneum, after which an 8 mm trocar is inserted as the camera port (C). A robotic arm operating port (R2) is created 5 cm to the right of the umbilicus at the level of the umbilicus (maryland bipolar forceps; 8 mm), while another robotic arm operating port (R1) is established 2 cm below the anterior axillary line on the left side (harmonic scalpel; 8 mm). Additionally, a third robotic arm operating port (R3) is positioned 2 cm below the anterior axillary line on the right side (non-invasive grasping forceps; 8 mm). An auxiliary operating port (A) is created 5 cm to the left of the umbilicus (12 mm) (**Figure 2A**). The patient is adjusted to a head-up, foot-down position of 15° to 30°, and the fourth-generation da Vinci robotic surgical system is introduced. The assistant is positioned on the left side of the patient (refer to the surgical layout diagram in **Figure 2B**).

#### 2.2.2 Abdominal Exploration

**Figure 3:**
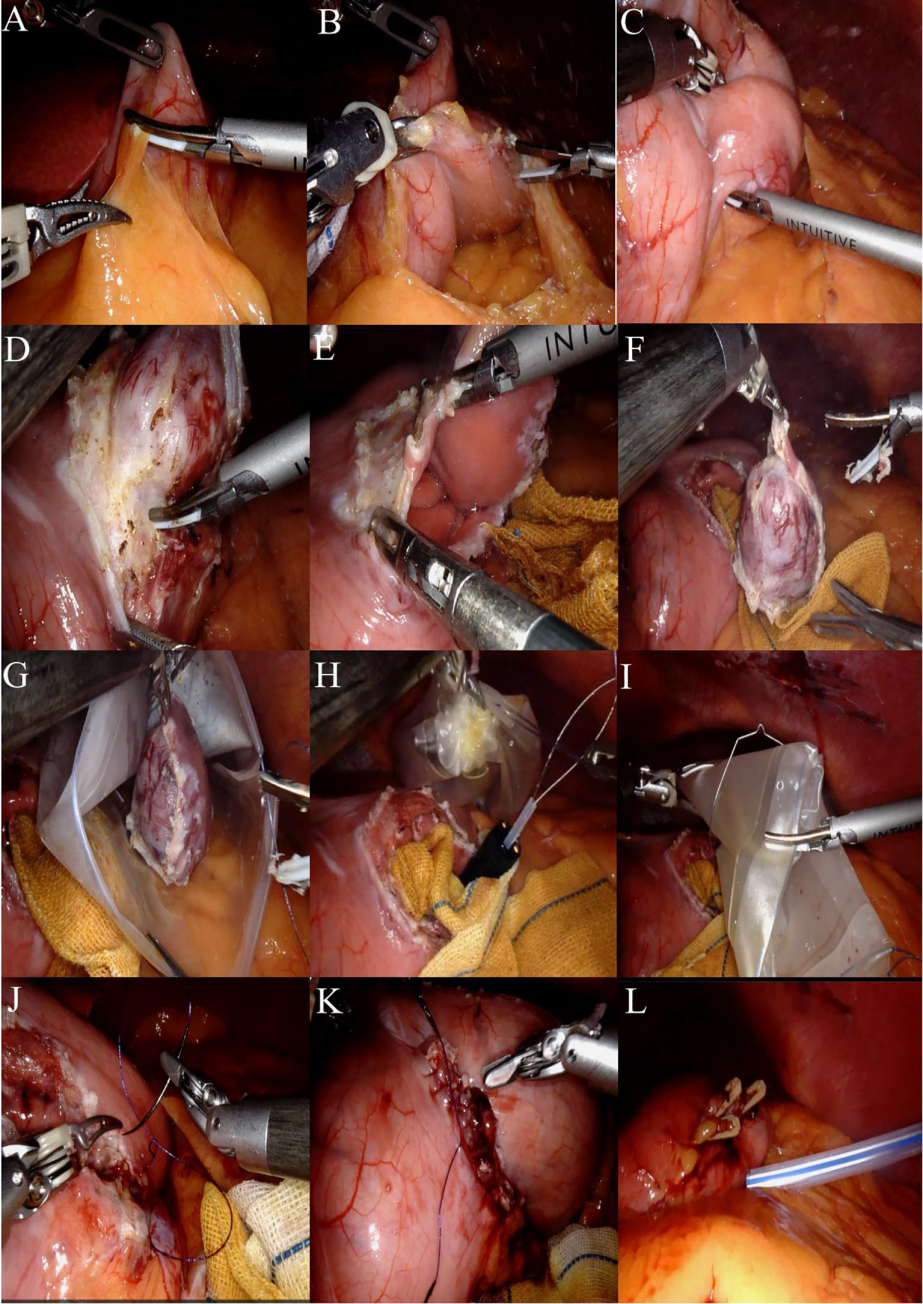
**(A)** The greater omentum was carefully dissected along the gastric wall. **(B)** The posterior gastric wall was fully exposed. **(C)** Upon exploration, a firm mass measuring approximately 4.0 cm × 3.5 cm × 3.0 cm was identified at the posterior wall of the lower gastric body near the lesser curvature, protruding into the gastric lumen. **(D)** After adequate exposure, the harmonic scalpel was used to meticulously dissect and isolate the tissue approximately 1 cm from the tumor margin. **(E)** The gastric wall was incised to access the gastric cavity. **(F)** The tumor was completely resected with a circumferential margin. **(G)** The specimen was placed into a retrieval bag. **(H)** An endoscopic retrieval device was introduced, and the specimen bag was securely grasped. **(I)** The robotic arm assisted in transferring the retrieval bag from the gastric wall incision into the gastric cavity. **(J)** The gastric wall incision was closed in a full-thickness manner using a 3–0 barbed unidirectional self-anchoring suture, proceeding from the superior to the inferior aspect. **(K)** A seromuscular layer closure was performed using the same 3–0 barbed suture, advancing from the inferior to the superior aspect. **(L)** A 24# irrigable drainage tube was placed posterior to the gastric wall.

A systematic exploration of the abdominal cavity is performed, assessing the liver, gallbladder, stomach, spleen, omentum, colon, small intestine, peritoneum, rectum, and pelvis to confirm the absence of metastasis or other lesions. Incise the greater omentum closely along the gastric wall while preserving the vascular arcade (**Figures 3A, B**), thereby adequately exposing the posterior wall of the stomach. Upon exploration, a firm mass measuring approximately 4.0 cm × 3.5 cm × 3.0 cm is palpable in the posterior wall of the lower body of the stomach, located near the lesser curvature, projecting into the gastric lumen (**Figure 3C**).

#### 2.2.3 Anatomical dissection

Following adequate exposure, the harmonic scalpel is meticulously used to delineate approximately 1 cm along the tumor margin until the gastric wall is incised into the gastric lumen (**Figures 3D, E**). Under direct visualization, the tumor is completely excised in a circumferential manner, and when encountering major blood vessels, absorbable clips are employed for hemostasis (**Figure 3F**).

#### 2.2.4 Specimen retrieval

The specimen bag is introduced into the abdominal cavity through the auxiliary access port, where the specimen is placed into the bag and the opening is secured (**Figures 3G, H**). A subsequent intraoperative gastroscopy is performed, utilizing the endoscopic retrieval device to grasp the specimen-containing bag (**Figure 3I**). The robotic arm is then employed to maneuver the retrieval bag through the gastric wall incision into the gastric cavity, allowing for the slow extraction of the specimen via the esophagus under direct visualization with the gastroscope (illustrated in **Figures 2C, D**). Intraoperative rapid pathology results indicate: gastric mass, spindle cell tumor, suggestive of GIST, with no tumor identified at the resection margins.

#### 2.2.5 Postoperative examination and drainage tube placement

**Figure 4:**
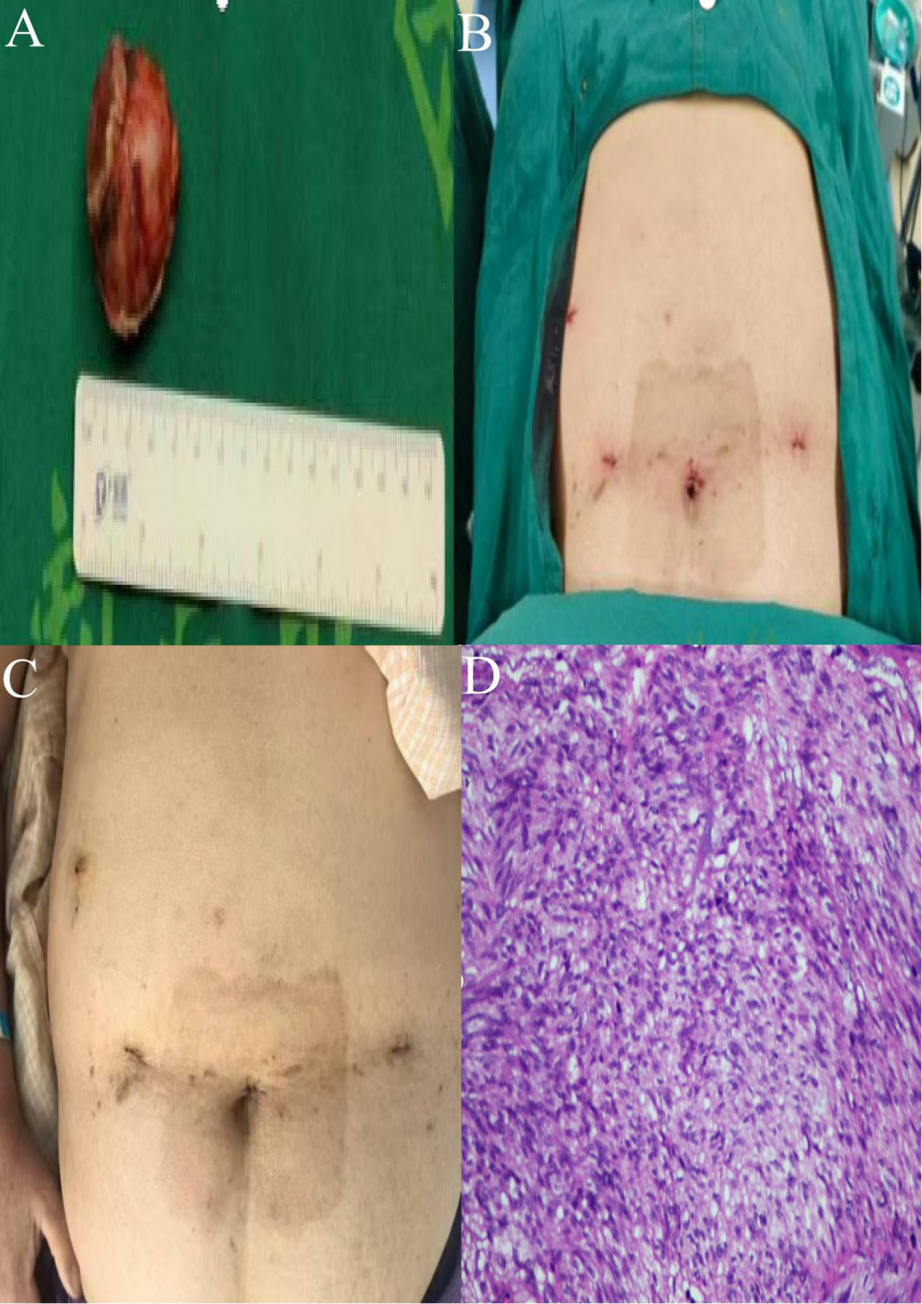
**(A)** Postoperative specimens from gastric GIST. **(B)** Abdominal wall incision post-surgery. **(C)** Healing status of the surgical wound. **(D)** Postoperative pathology (HE high magnification).

The surgical area was cleaned with iodine gauze, followed by full-thickness suturing of the gastric wall incision using a 3–0 self-fixing, knotless suture from the superior to the inferior aspect (**Figure 3J**). A second 3–0 self-fixing, knotless suture was then employed to encapsulate the gastric wall incision from the inferior to the superior direction through the muscular layer, secured with an absorbable clip to prevent suture displacement, and the suturing was assessed for adequacy (**Figure 3K**). A subsequent gastroscopy revealed no bleeding at the gastric margin, with the gastric lumen remaining patent and no injuries to the esophagus or oral cavity. A single drainage tube was placed posterior to the gastric wall and secured to the left operative port (**Figure 3L**). Instruments were counted, the abdominal incision was sutured, and the procedure was concluded. A comprehensive overview of the surgical process is illustrated in **Figure 3**. Postoperative specimens and the abdominal incision are depicted in **Figures 4A, B**.

### 2.3 Postoperative Condition of the Patient

The patient successfully underwent robotic-assisted resection of a GIST combined with a gastroscopy-assisted transoral biopsy (GC-NOSES IX). Intraoperative blood loss was approximately 5 mL, and the surgical duration was 320 minutes. The patient exhibited a favorable postoperative recovery, being able to ambulate and have the urinary catheter removed upon regaining consciousness from anesthesia. Flatus was noted on postoperative day 1, with attempts to drink water initiated thereafter. On postoperative day 2, the patient began a trial of a clear liquid diet, and the drainage tube was removed on postoperative day 7, allowing for safe discharge on postoperative day 9. The incision site demonstrated satisfactory healing, and no complications were observed postoperatively (**Figure 4C**). The pathological findings revealed: (1) Gross examination: a mucosal tissue specimen from the gastric mass measuring 7.5 cm × 2.5 cm^2^, with a submucosal nodular lesion measuring 4.0 cm ×3.0 cm×3.0 cm. (2) Pathological diagnosis: GIST measuring 4.0 cm × 3.0 cm × 3.0 cm, exhibiting mild to moderate cellular atypia, with a mitotic index of 3 per 5 mm², classified as low risk, with no tumor identified at the resection margins. Immunohistochemistry results were as follows: CD117 (+), CD34 (+), Desmin (-), DOG-1 (+), Ki67 positivity in approximately 5% of tumor cells, S-100 (-), SDHB (+), SMA (few cells +), SOX-10 (-) (**Figure 4D**).

Based on the postoperative pathological findings, the patient is diagnosed with a non-SDHB-deficient GIST, classified as low-risk. This suggests a favorable prognosis, indicating that adjuvant targeted therapy is unnecessary. Instead, regular postoperative follow-up is recommended, with the following surveillance schedule: enhanced CT or MRI of the abdomen and pelvis every 6–12 months for the first 3–5 years post-surgery. After 5 years, the frequency can be reduced to once annually. Should recurrence or metastasis occur in the future, targeted therapy (such as imatinib) or surgical intervention may be considered as appropriate (10).

## 3 Discussion

With the rapid advancement of minimally invasive surgical techniques, Natural Orifice Specimen Extraction Surgery (NOSES) has evolved from its nascent stages to a more refined practice. An international consensus on transnatural orifice specimen extraction for gastric neoplasms is progressively being formulated (9). In gastric NOSES, the primary pathways for specimen retrieval are the transanal and transvaginal routes. Furthermore, certain researchers have investigated the feasibility of transoral specimen extraction in NOSES procedures, utilizing both animal models and clinical patients. This approach has been applied in surgeries such as cholecystectomy, sleeve gastrectomy, hepatic cystectomy, adrenal mass excision, early gastric tumor resection, and leiomyoma excision (11–17). The viability of transoral specimen retrieval for GISTs has been substantiated by pertinent scholarly publications (18, 19). In transoral NOSES, the esophagus serves as the sole conduit for specimen retrieval, endowing this procedure with distinct uniqueness. Compared to the rectum and vagina, the esophagus presents a narrower and less elastic lumen, which heightens the complexity of the operation and imposes stricter criteria for specimen extraction. Surgeons must rigorously adhere to the principles of “asepsis and tumor-free” and possess proficient mastery of NOSES surgical protocols. Typically, the indications for transoral NOSES in gastric tumors include: ① Gastric wall neoplasms, either benign or malignant, that are not amenable to complete endoscopic resection; ② lesions ideally measuring less than 2.0 cm in maximum diameter; ③ appropriate for tumors classified as T2 or T3 stage. Relative contraindications include: ① neoplasms with advanced local staging and larger dimensions; ② patients with obesity (body mass index ≥30.0 kg/m²); ③ individuals experiencing acute gastrointestinal obstruction, tumor perforation, or hemorrhage necessitating urgent surgical intervention (9). Owing to its distinctive characteristics, there remains a scarcity of scholarly work on this topic, especially regarding robot-assisted gastric GIST resection with endoscopic transoral specimen extraction (GC-NOSES type IX). In this case, the patient is an elderly female with a mass located on the posterior wall of the lower body of the stomach. She has a strong preference for minimally invasive surgery and has no prior history of oral or esophageal diseases or surgeries. The moderate size of the mass provides feasibility for surgical intervention. Following a preoperative discussion, we identified several surgical options: robotic-assisted resection of the GIST combined with endoscopic transoral specimen retrieval, non-exposed endoscopic wall-inversion surgery (NEWS), and traditional laparoscopic surgery. After extensive deliberation, we concluded that: ① The patient’s BMI >30 kg/m², which complicates endoscopic inversion; the tumor is relatively large and situated on the posterior wall of the lower stomach near the lesser curvature. If NEWS were employed for resection, the limited endoscopic working space and suboptimal visualization would complicate the procedure, increasing the risk of excessive manipulation and potential tumor rupture, thereby jeopardizing the possibility of achieving an R0 resection. ② Literature indicates that even experienced operators may require over five hours to complete a NEWS procedure (20). Based on our previous experiences, we believe that this technique is time-consuming and may hinder postoperative recovery, while also increasing the risk of symptoms such as throat discomfort and complications. ③ The NEWS technique poses challenges in achieving precise apposition of the serosal layer during suturing, with a risk of delayed perforation. Additionally, closure of the mucosal defect under endoscopic guidance necessitates the use of clips, which can delay resumption of oral intake. ④ Our approach integrates the advantages of the Da Vinci robotic surgical system with endoscopy. Compared to NEWS and traditional laparoscopic surgery, it offers several benefits: ① The Da Vinci system allows for precise and flexible manipulation, enabling access to areas that are difficult to reach with conventional endoscopy, facilitating complete tumor resection and improving the R0 resection rate. ② It can reduce operative time and lower the incidence of complications; furthermore, suturing is more meticulous and accurate, resulting in less damage to the gastric wall and quicker recovery of gastrointestinal function, allowing for early resumption of feeding. ③ During the transoral specimen retrieval, we can accurately assist in placing the specimen into a retrieval bag, ensuring a completely non-exposed procedure to prevent tumor cell seeding.④ While adhering to the principle of tumor-free margins, we also strive to minimize gastric fluid spillage to avoid contamination of the gastric cavity, employing iodine-impregnated gauze strategically. ⑤ The implementation of this technique is based on a comprehensive assessment to ensure surgical safety and facilitate patient recovery. By overcoming the traditional dilemma of tumor size versus minimally invasive surgery, we have achieved a one-stop solution that encompasses radical treatment, aesthetic considerations, and rapid recovery. Consequently, we decided to proceed with robotic-assisted resection of the gastrointestinal stromal tumor combined with endoscopic transoral specimen retrieval. Following successful anesthesia, we conducted a thorough endoscopic evaluation of the patient’s esophageal condition, determining that her esophageal elasticity was relatively good, with a patent lumen, thus confirming the feasibility of the procedure. The specimen retrieval was performed under continuous visualization and proceeded smoothly; upon completion, a follow-up endoscopic examination revealed that the esophagus and oral cavity were intact without any damage.

This technique obviated the necessity for enlarging the abdominal incision, thereby minimizing incision-related complications and postoperative discomfort such as pain, which facilitated recovery and reduced hospitalization expenses. The operation duration was 320 minutes with an intraoperative blood loss of approximately 5 mL. The procedure adeptly combined the benefits of the Da Vinci robotic surgical system and endoscopy, markedly improving the patient’s overall postoperative quality of life.The patient exhibited a favorable postoperative recovery, demonstrating the ability to ambulate and have the urinary catheter removed upon regaining consciousness from anesthesia. Flatus was noted on postoperative day one, followed by attempts at oral hydration. On postoperative day two, the patient commenced a trial of a clear liquid diet, with the drainage tube being removed on postoperative day seven. The patient was discharged safely on postoperative day nine. There were no complications observed post-surgery, the incision site healed appropriately, and the patient expressed high satisfaction with the care received.

Based on our department’s substantial experience with more than 500 robot-assisted gastrointestinal surgeries and dual-endoscope techniques, notably the pioneering robot-assisted gastric stromal tumor resection with endoscopic transoral specimen extraction (GC-NOSES IX), we have gleaned the following insights:

1. In transoral NOSES procedures, the esophagus serves as the essential conduit for specimen retrieval. Compared to the rectum and vagina, the esophagus is more elongated and has less elastic wall properties. Additionally, the esophagus features three physiological constrictions, which can predispose to mucosal injury and tearing hemorrhage during specimen extraction, particularly in the presence of esophageal varices, where intraoperative esophageal bleeding is more likely to occur. In our case, the maximum diameter of the specimen was 4.0 cm, and the extraction process was relatively smooth, with no damage to the esophageal or oral mucosa. It is generally accepted that a specimen diameter of less than 3.0 cm is preferable for transoral retrieval. Based on previous literature and our experience, where the maximum diameter of specimens retrieved transorally has reached 6.25 cm (15), we assert that the esophagus is indeed a critical pathway for specimen extraction, necessitating stringent size requirements. Therefore, preoperative assessment of indications must be conducted with utmost caution and rigor. If the specimen size is appropriate and the patient’s esophageal condition is favorable, this technique may be attempted under safe and meticulously controlled circumstances. For patients with higher risk factors, this approach is not recommended.
2. According to Gündoğan et al. (21), NOSES procedures carry a risk of tumor seeding. In transoral NOSES, since a specimen protection device cannot be pre-placed in the esophageal lumen, it is crucial to enclose the specimen in a sealed retrieval bag before esophageal extraction to ensure tumor-free operation.
3. Due to the intraoperative perforation of the gastric cavity, it is recommended to adequately prepare the patient’s gastrointestinal tract preoperatively, ensure sterilization of the gastroscope, administer prophylactic antibiotics prior to the procedure, and utilize iodine-impregnated gauze for disinfection of the surgical field during the operation to achieve aseptic conditions as much as possible.
4. Intraoperative tracheal intubation may adversely affect specimen retrieval. If resistance is encountered during extraction, it is imperative to avoid forceful traction to prevent esophageal wall injury. Utilizing gastroscopy assistance, the specimen should be retrieved under direct visualization through the esophagus to maximize procedural safety.
5. In cases where intraoperative tumor localization proves difficult, endoscopic assistance may be utilized to facilitate dual-endoscope localization and surgical intervention. Additionally, endoscopy serves to confirm the precision of suturing, assess gastric lumen constriction, and detect hemorrhage.
6. Drawing from our center’s extensive experience with more than 500 fourth-generation Da Vinci robotic gastrointestinal procedures, as well as corroborating literature (20), it is evident that intraoperative robotic malfunctions can arise, underscoring the need for readiness in handling such emergencies.
7. Tumor excision can be executed via two techniques: wedge resection utilizing a linear stapler, necessitating a more extensive tissue excision and heightening the risk of postoperative gastric constriction (22), or local wedge resection employing an ultrasonic scalpel with subsequent continuous suture closure, selected according to the tumor’s anatomical position.
8. The Da Vinci robotic surgical system and endoscopic transoral specimen extraction demand a high level of specialization, requiring endoscopists to possess exceptional technical expertise. Comprehensive preoperative planning, collaborative teamwork, and meticulous intraoperative coordination are vital for successful outcomes. It is advised that endoscopists within the surgical team attain proficiency in digestive endoscopy knowledge and techniques.

In summary, the integration of robot-assisted gastric GIST resection with endoscopic transoral specimen extraction (GC-NOSES IX) offers notable advantages in minimally invasive surgery. Nevertheless, given the esophagus’s distinct anatomical configuration, it is imperative for surgeons to meticulously determine surgical indications, refine operative skills, and foster robust team collaboration to optimize patient outcomes and maintain surgical safety.

## Data Availability

The original contributions to the study are detailed within the article/supplementary materials. Any further queries should be addressed to the corresponding authors.
